# Atomic force microscopy analysis of nanoparticles in non-ideal conditions

**DOI:** 10.1186/1556-276X-6-514

**Published:** 2011-08-30

**Authors:** Petr Klapetek, Miroslav Valtr, David Nečas, Ota Salyk, Petr Dzik

**Affiliations:** 1 Czech Metrology Institute, Okružní 31, 638 00, Brno, Czech Republic; 2 Department of Physical Electronics, Faculty of Science, Masaryk University, Kotlářská 2, 611 37 Brno, Czech Republic; 3 Faculty of Chemistry, Brno University of Technology, Purkyňova 118, 612 00 Brno, Czech Republic

## Abstract

Nanoparticles are often measured using atomic force microscopy or other scanning probe microscopy methods. For isolated nanoparticles on flat substrates, this is a relatively easy task. However, in real situations, we often need to analyze nanoparticles on rough substrates or nanoparticles that are not isolated. In this article, we present a simple model for realistic simulations of nanoparticle deposition and we employ this model for modeling nanoparticles on rough substrates. Different modeling conditions (coverage, relaxation after deposition) and convolution with different tip shapes are used to obtain a wide spectrum of virtual AFM nanoparticle images similar to those known from practice. Statistical parameters of nanoparticles are then analyzed using different data processing algorithms in order to show their systematic errors and to estimate uncertainties for atomic force microscopy analysis of nanoparticles under non-ideal conditions. It is shown that the elimination of user influence on the data processing algorithm is a key step for obtaining accurate results while analyzing nanoparticles measured in non-ideal conditions.

## Introduction

Nanoparticle analysis is an important challenge in the present nanoscale metrology. Nanoparticles are used in many fields of research and technology [1-5], and their proper characterization is, therefore, very important. Even if there are several general and well established experimental methods to nanoparticle analysis (optical methods [6-8], electrochemistry-based methods [9], electron microscopy [10,11], X-ray methods [10,12] and scanning probe microscopy [10,13]), their results differ mutually very often due to different effects of non-ideal measurement conditions [6,7,10,14].

In this article, we focus on nanoparticle analysis performed using atomic force microscopy (AFM) [15], which is one of the most popular scanning probe microscopy methods. The interaction of nanoparticles with the AFM probe was studied in the past quite extensively from the experimental point of view--from the point of nanoparticle measurement, AFM tip modification, or nanoparticle manipulation [10,13,16-18]. If the isolated nanoparticles of spherical shape are deposited on an ideally flat substrate, their size can be determined easily from the AFM image by measuring the nanoparticle image height [13]. This quantity is not influenced by tip-sample convolution effects and can provide accurate nanoparticle size results.

However, if the particles are deposited on rough substrates (or curved substrates generally), particle size analysis is not so straightforward and therefore many questions arise from the point of particle analysis implementation in AFM image processing software. Another effect strongly influencing the AFM analysis of nanoparticles is particle agglomeration and self-ordering on the substrate. In real measurements, we can often observe both effects. The statistical results of nanoparticle properties therefore rely on a good choice and correct use of AFM data evaluation algorithms, which adds a human error to the whole measurement process. From a metrology point of view, this approach is not satisfactory, as we cannot easily determine the measurement uncertainty.

The aim of this article is to investigate the influence of the substrate roughness and particle agglomeration on the statistical analysis of nanoparticle properties. We study the measurement uncertainty of nanoparticle parameters with respect to different nanoparticle data processing methods and scanning parameters (e.g., tip related effects). To do this, we employ a simple model that simulates the real particle deposition and ordering on the substrate and the basic physical phenomena connected with this processes. Afterward, we simulate AFM scans obtained on modeled samples and we evaluate nanoparticle statistical properties using different data analysis methods. Finally, we compare the results with the nominal values of the nanoparticle statistical properties used for modeling on the first stage. This approach enables us to determine the level of confidence in AFM measurements of nanoparticles and to determine the limits of measurement uncertainty in these cases.

## Experimental arrangement

**Figure 1:**
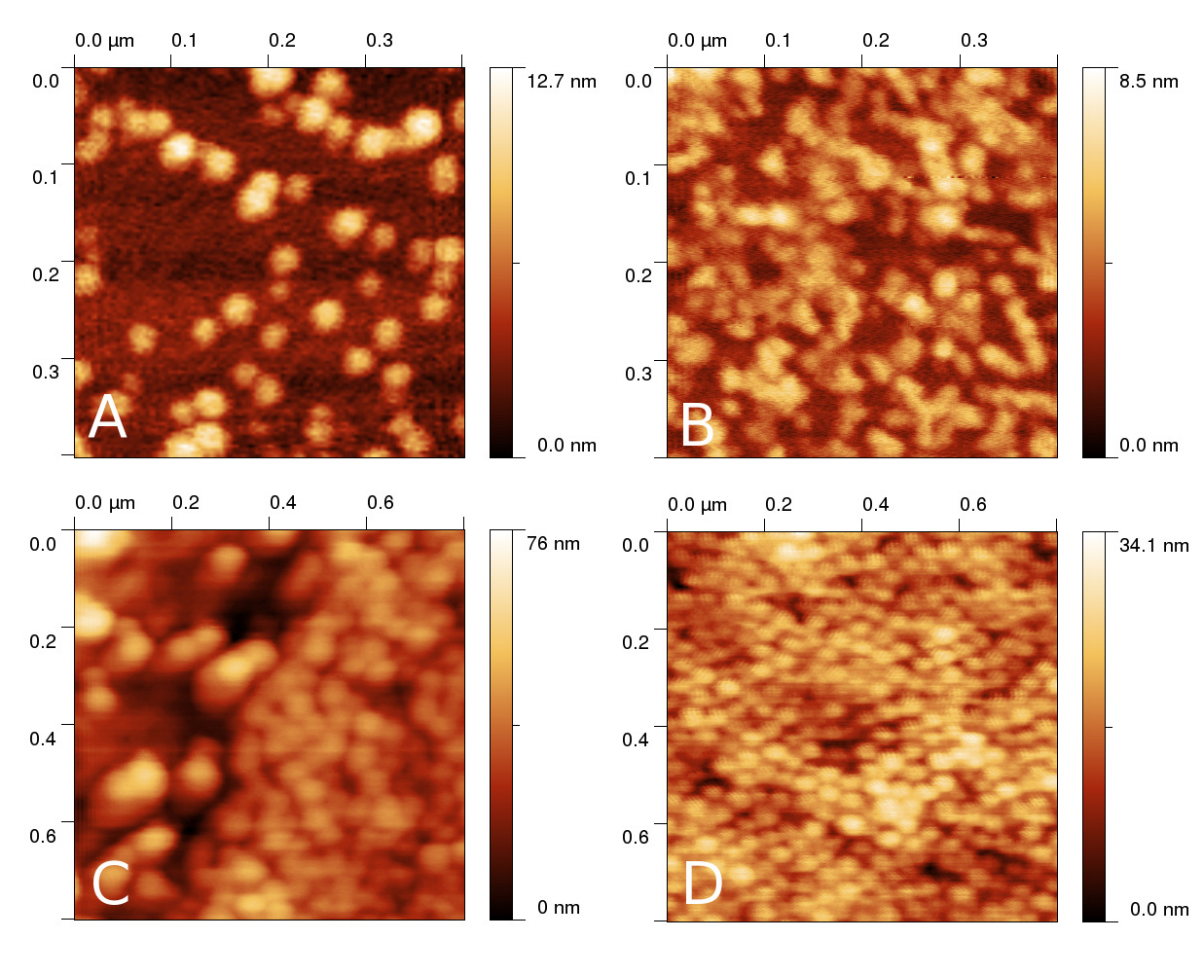
**Typical AFM nanoparticle measurement of palladium on flat (a) and rough silicon (b), polymer on rough (c) and flat silicon (d)**.

Atomic force microscopy measurements shown in Figure 1a-d to illustrate the numerical models connection to real data in this article were performed using AFM Explorer (Thermomicroscopes) in contact and non-contact mode, using standard contact (type MSCT-EXMT-A1) probes supplied by Veeco company and non-contact probes (type PPP-NCLR) supplied by NanoAndMore company. Measurements were performed in ambient conditions. Image resolution was between 500 × 500 pixels and 1, 000 × 1, 000 pixels, scan speed between 1 and 5 *μ*m/s. Raw data obtained from the microscope were processed in Gwyddion open source software using the plane leveling algorithm [19].

Nanoparticle samples were prepared by spin-off coating, using a simple home-built apparatus; nanoparticles were dispersed in ethylene glycol [20] (palladium) resp. in water (polymer) and dried after deposition.

## Data modeling and analysis

In order to simulate the full process of nanoparticle deposition, measurement and analysis, data modeling was performed in several successive steps:

1. modeling of a **rough surface**.

2. simulation of **particle deposition **on the surface.

3. creation of virtual AFM images by **tip-sample convolution**.

4. nanoparticle **statistical analysis **using virtual AFM images and data processing software.

In order to simulate the effects of both surface roughness and nanoparticle clustering, we need to vary the following parameters in steps 1 -3:

• surface roughness (parametrized using the root mean square roughness and autocorrelation length),

• number of particles and their size (parametrized using the surface coverage and the particle radius),

• AFM tip shape (parametrized using the tip radius and the apex ratio).

The resulting nanoparticle statistical properties are then compared to values used in step 2 (particle deposition). The algorithms used for data modeling are described in more detail in the next two sections. All the data modeling and processing algorithms were implemented in Gwyddion open source software http://gwyddion.net and are available for public in the present version of software.

### Surface generation and particle deposition modeling

**Rough substrates **were modeled to have a Gaussian autocorrelation function [21], which is a simple model often used in many fields of surface physics [22].

First, a sufficiently large field filled by independent random numbers needs to be created, having these properties:

(1) ⟨η(r)⟩=0

(2) $$\left\langle \eta \left(r\right)\eta \left(r^{\prime }\right)\right\rangle =\pi^{d∕2}\delta \left(r-r^{\prime }\right).$$

where *η *(**r**) is the random number at the position given by **r**, *η *(**r**') is the random number at the position given by **r**' and *d *is the surface dimensionality (here *d *= 2).

The surface *z*(**r**) is created by performing a convolution according to the formula

(3) $$z\left(r\right)=2^{d∕2}\frac{\sigma T^{-d∕2}}{\pi^{d∕2}}\overset{\infty }{\underset{-\infty }{\int }}\exp\left[-2\frac{{\left(r-r^{\prime }\right)}^{2}}{T^{2}}\right]\eta \left(r\right)\text{d}r^{\prime },$$

where *σ *and/or *T *are the root mean square roughness and/or autocorrelation length corresponding to the surface to be constructed. As this integral is evaluated numerically, it is necessary to limit the computation to a sufficient area, depending on the decay of the Gaussian function inside the integral. The resulting values *z*(**r**) form a surface with the required root mean square roughness *σ *and autocorrelation length *T*.

Surface properties are therefore controlled by two parameters--the root mean square roughness (*σ*) and the autocorrelation length (*T*). Note that for our simulation, the autocorrelation length was kept constant and only the root mean square roughness was varied.

For **particle deposition**, a simple model similar to molecular dynamics calculations was constructed as described below. The aim of the model is to include basic interaction between nanoparticles and between a nanoparticle and the substrate and to model the effects of thermal and mechanical vibrations in nanoparticle dispersion (e.g., Brownian motion). In contrast to more rigorous models shown in literature [10,23], this model does not include the effects of nanoparticle atomistic structure or effects of the presence of the vapor phase [24]; however, as seen from the results, it is still able to generate images of nanoparticles very similar to real images observed using atomic force microscopy (see Figure 1a-d). In contrast to even simpler models (e.g., random placement of nanoparticles on substrate), it can include the nanoparticle self-organization effects, which are important phenomena affecting nanoparticle analysis in scanning probe microscopy as seen in Figure 1c-d.

In order to model the nanoparticle deposition, we used the following algorithms and physical models:

• Nanoparticles were modeled as Lennard-Jones spheres, the surface by an integrated Lennard-Jones potential [25,26].

• Verlet algorithm was used for the integration of the Newton equations.

• The Anderson thermostat was used to simulate Brownian motion of nanoparticles in a liquid (which is the nanoparticle deposition in practice).

• Nanoparticle velocities were damped during computation to simulate the decreasing mobility.

**Figure 2:**
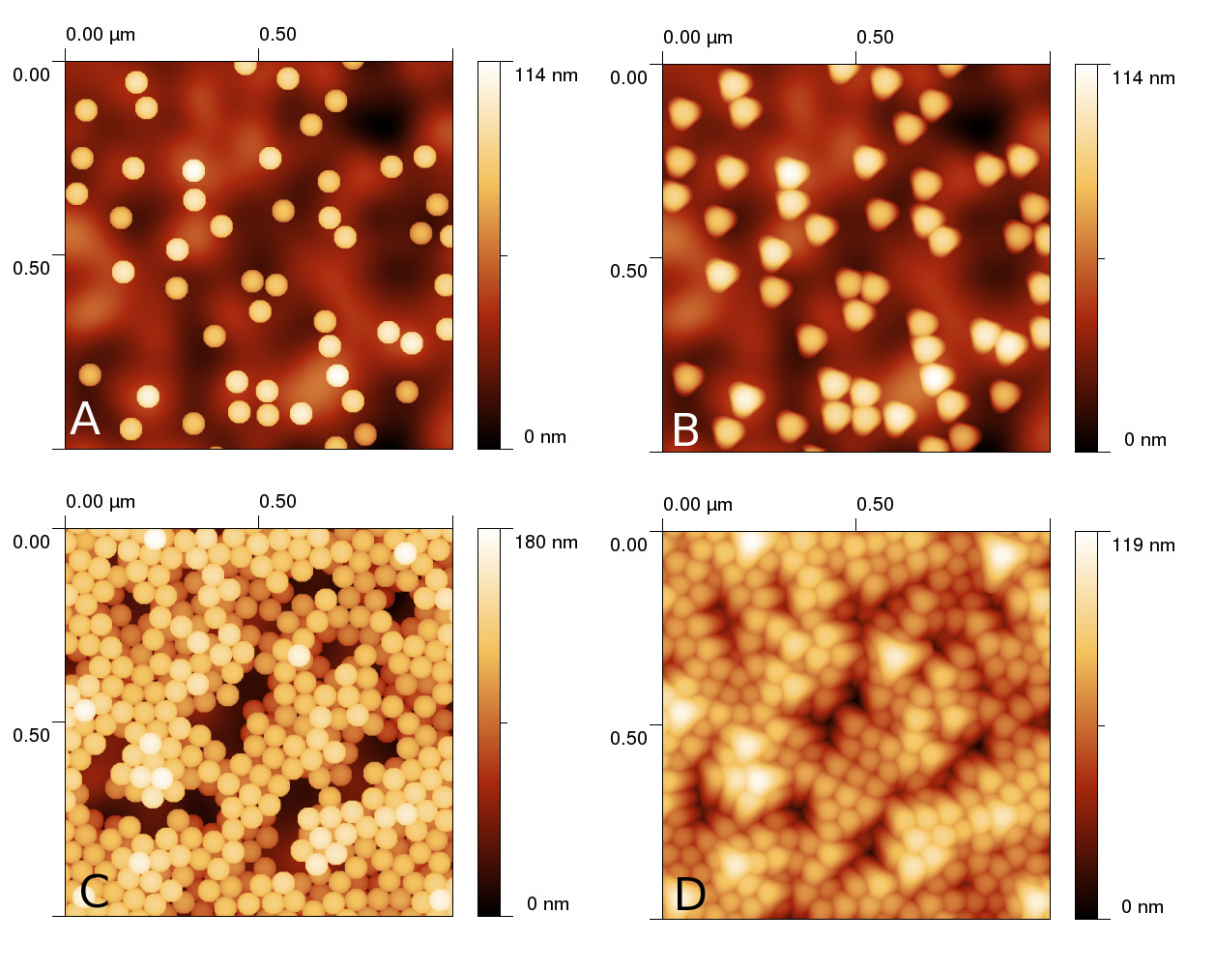
**Results of nanoparticle deposition modeling and tip convolution**: **(a)**--isolated nanoparticles with no convolution (or convolved with tip 1), **(b)**--isolated nanoparticles convolved with tip 2, **(c)**--film of nanoparticles with no convolution (or convolved with tip 1), **(d)**--several layers of nanoparticles convolved with tip 3.

Typical images of nanoparticles obtained using this approach are given in Figure 2a, c. The developed algorithm enables us to create even more complex structures by varying the particle number, mobility and force constants between particles and between particle and substrate. A structure similar to a real measurement can be therefore obtained relatively easily even for other samples than those shown in Figure 1a-d.

### Virtual microscopy measurement and data evaluation

**Figure 3:**
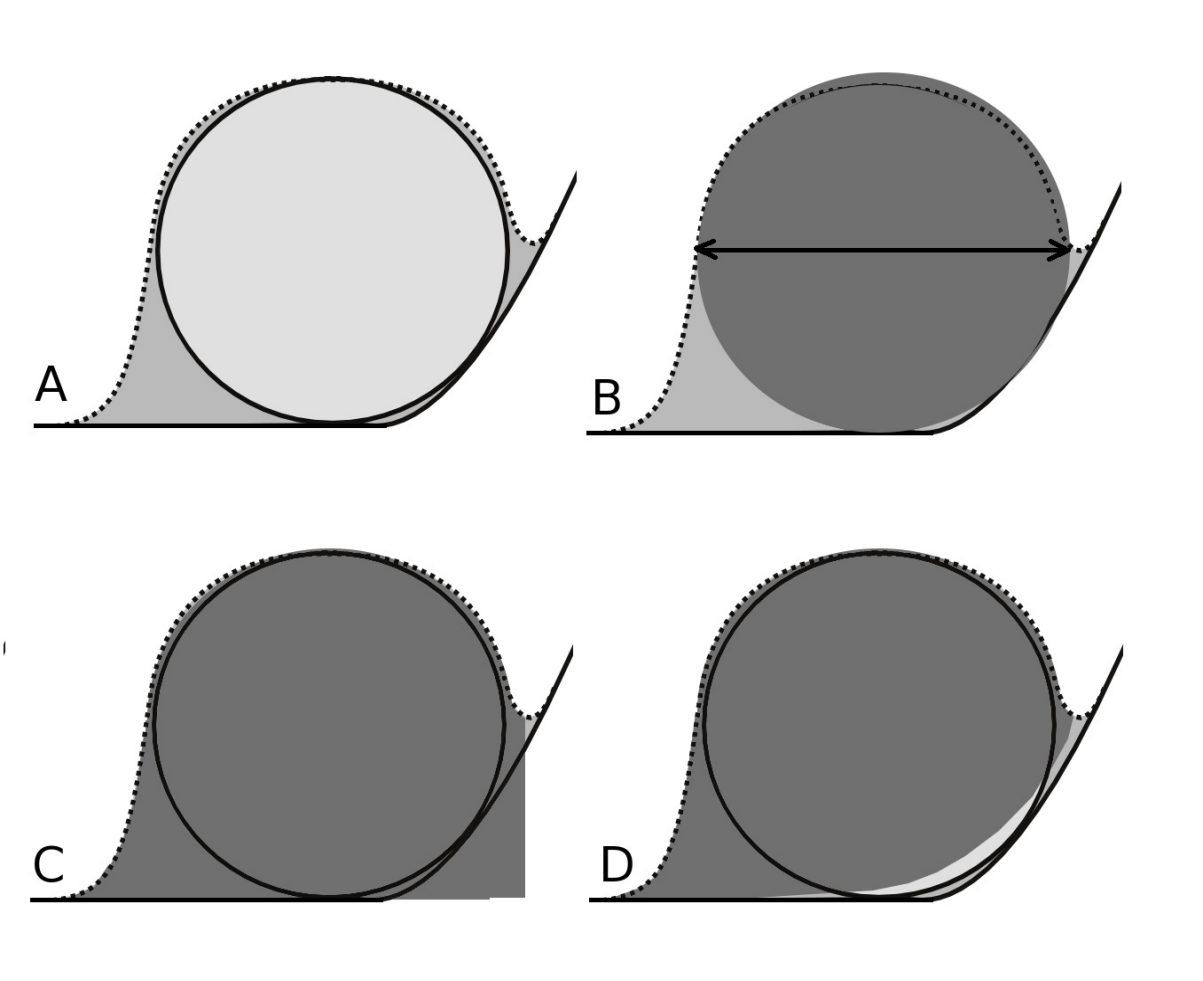
**Schematics of data processing methods**. Nanoparticle on curved substrate **(a)**. Solid lines denote the substrate and the nanoparticle, respectively, and the dotted line denotes the path of the AFM tip. The convolution effect on used data processing algorithms: **(b)**--nanoparticle projection, **(c)**-- nanoparticle volume at minimum basis, **(d)**--nanoparticle volume at Laplacian basis. Light gray represents the nanoparticle. Medium gray shows the nanoparticle as seen by AFM (after tip convolution), dark gray represents volume determined by the data processing algorithm. Note that even Laplacian basis cannot properly determine the whole nanoparticle volume as the surface geometry below nanoparticle is unknown. As the power spectrum-based algorithm is a global one, it cannot be illustrated within this schematic figure.

Virtual AFM measurement were performed using the dilation algorithm presented in Ref. [27]. As we focused on the analysis of statistical data processing methods in this article, we limited the selection of used AFM tips to a few commercially available probes (described by their nominal radius and aspect ratio parameters). The dilation algorithm returns simulated AFM images, determined morphologically as a convolution of rigid bodies. This approach is therefore valid under the assumption that there are no tip or sample changes due to tip-sample interaction. This could be in principle problematic for very soft materials or for extremely small nanoparticles whose geometry could change significantly due to tip-sample forces [28,29]. Examples of results of the dilation on simulated surfaces are given in Figure 2b, d. We can see that the resulting images are very similar to the real measurements (shown in Figure 1a-d). The effect of dilation on a single nanoparticle on a curved substrate is shown in Figure 3a. We can see that the tip convolution prevents the AFM from seeing the morphology below the nanoparticle. As there is no complete information about both nanoparticle and substrate geometry at these parts of AFM image, all the data processing algorithms need to make some assumptions regarding nanoparticle and substrate properties.

For the characterization of nanoparticles from AFM data, several algorithms can be used. The first stage is always the segmentation of data into separate particles. In principle, a simple thresholding can be used to do this for the case of isolated particles on a flat substrate. For rough or curved substrates and for agglomerated particles, this approach usually fails as we cannot determine an appropriate threshold value; therefore, we use a watershed approach for image segmentation in this article.

The watershed algorithm is a relatively simple alternative to thresholding, providing much better segmentation on complex structures [30,31]. In this case, AFM data are first inverted in the *z*-direction. Virtual drops are then placed on the surface, leaving them to relax to minimum height, forming small "lakes." This is the first place (so-called location phase) after which the individual "lakes" are associated with a particle. After that, in the second phase (segmentation phase) the drops continue to be placed on the surface and relaxed, but unlike during the first phase, they are no longer allowed to merge. This leads to image segmentation. As a result, we obtain an image with marked individual grains.

The second stage is to convert marked grains into grain size distribution information of the nanoparticle characterization. We can divide the nanoparticle processing algorithms into three categories:

1. algorithms based on nanoparticle projection on the *xy *plane. Even if this quantity can be highly affected by tip convolution, the effect of substrate curvature or particle agglomeration can be much smaller here than for height-based algorithms. In this paper, this class of algorithms is represented by equivalent disk projection.

2. algorithms based on nanoparticle height or volume determination. For flat substrates and non-agglomerated particles, this class of algorithms leads to results that are not affected by tip-sample convolution effects. For curved substrates, they can differ in the treatment of the surface beneath the particle, which cannot be observed using the AFM. In this paper, this class of algorithms is represented by two methods--volume analysis using minimum boundary and Laplacian boundary method.

3. algorithms based on the statistical analysis of the whole measurement, e.g., autocorrelation function or power spectral density. These algorithms benefit from the self-organization of nanoparticles on substrate; in an ideal case, a honeycomb thin film is observed. In this work, power spectral density method is used.

There can be many varieties of details of the mentioned algorithms, providing slightly better or worse results in particular cases. In this article, we cannot discuss all of them. We aim to show the main trends in errors of different classes of algorithms under non-ideal conditions in order to estimate how much we can trust their results.

For nanoparticle radii evaluation, the following algorithms were used (all based on their Gwyddion implementation, see http://gwyddion.net):

• particle radius determined on the basis of equivalent disks (having the same area as the *xy *projection of particle). Here, the selected particle area (obtained from image segmentation, see above) is directly used as the particle cross-section. Particle volume is then calculated as volume of sphere having the same cross-section (see Figure 3b).

• particle radius determined on the basis of its volume with respect to its boundary minimum. Here, the particle boundary is obtained from image segmentation, and the minimum height value along this boundary is used as a lower boundary to measure the particle volume (see Figure 3c). A numerical correction by a factor of 0.8 is employed to remove the apparent volume below the particle due to projection. The factor of 0.8 is the ratio between sphere volume and the volume of the sphere together with its cylindrical projection to the substrate.

• particle radius determined on the basis of its volume with respect to Laplacian interpolation of its boundary. Here, the particle boundary is obtained by image segmentation, and Laplacian interpolation is run to obtain the morphology of the substrate below the particle (see Figure 3d). A numerical correction by a factor of 0.8 is employed to remove the apparent volume below particle due to projection.

• particle radius determined by radial power spectral density evaluation. Here, a 2D Fast Fourier transform is performed from the whole image, and the particle size is determined from the observed maxima in the resulting radial power spectrum, which in the ideal case of closely packed nanoparticles are directly connected with the size of the nanoparticle.

As shown in the next section, each of the algorithms has its own benefits and drawbacks with respect to the treatment of nanoparticle agglomeration, tip size, or surface roughness.

## Results and discussion

Typical examples of nanoparticle measurements are shown in Figure 1a-d, representing palladium and polymer nanoparticles of different surface coverages, deposited on a flat silicon and a rough (anodically etched) silicon surface. We can see that both substrate roughness and particle agglomeration can be easily seen on the AFM images.

We have simulated several sets of nanoparticles on rough substrates with variable roughness (*σ *= 0 ÷ 10 nm). This range was chosen in order to include typical surface morphologies observed on surfaces and thin films prepared by different technological methods [32,33]. Note that the surface root mean square value of 10 nm represents surface morphology with minimum to maximum range of some 100 nm, which is already a very high value (higher than the simulated nanoparticle size).

First, the nanoparticle coverage (ratio of sample area occupied by particles to total sample area) was varied, to include all the typical effects starting from isolated nanoparticles up to a substrate covered by several layers of nanoparticles. Secondly, the effects of relaxation and self-ordering of nanoparticles were studied, simulating the nanoparticles with same coverages but different mobility and relaxation parameters during deposition modeling.

In the following paragraphs, the effects of tip convolution on different nanoparticle processing and evaluation algorithms are discussed. For analysis, we have chosen three different AFM tips:

1. ideal tip, represented by a *δ*-function (0 nm tip radius and slope of 90°), unavailable in practice but sometimes almost reached by carbon nanotubes - based tips [14,34].

2. sharpened tip with 10 nm tip radius and slope of 75°.

3. standard tip with 15 nm tip radius and slope of 57°.

In Table 1, results for the chosen data evaluation algorithms are presented. Values are calculated for different coverages (13, 50 and 140%), different AFM tips and different roughnesses (*σ *= 0 and 10 nm). A nominal particle radius of 30 nm was used for the modeling, representing a value in the mid-range of the typical reference nanoparticles sizes. The maximum in the size distribution or in the power spectrum (determined using one of the algorithms mentioned above) was used to determine the mean nanoparticle size. Presented uncertainties are based on widths of appropriate distributions, so they do not contain any systematic error or other B-type uncertainty information [35]. We can see that for a flat substrate and an ideal tip, we get the nominal values (which could be expected); for a rough substrate or non-ideal tip values, we can see increasing differences between nominal values and results. Note that for some cases, there was no maximum observed in appropriate distribution and results could not be obtained.

**Table 1:** Nanoparticle radii results of nanoparticle radii simulated measurements for nanoparticles with nominal radius of 30 nm and different surface coverages.

		*σ *= 0 nm				*σ *= 10 nm		
	**Pow**	**Min**	**Lap**	**Disk**	**Pow**	**Min**	**Lap**	**Disk**

C1, tip 1	32 ± 2	30 ± 1	30 ± 1	30 ± 1	33 ± 1	30 ± 3	30 ± 1	30 ± 1
C1, tip 2	37 ± 7	34 ± 1	34 ± 1	44 ± 2	44 ± 20	33 ± 5	32 ± 4	41 ± 3
C1, tip 3	N. A.	33 ± 3	33 ± 3	52 ± 5	N. A.	34 ± 6	34 ± 5	55 ± 9
C2, tip 1	32 ± 1	30 ± 1	30 ± 1	29 ± 1	33 ± 2	30 ± 2	30 ± 1	30 ± 1
C2, tip 2	38 ± 5	29 ± 3	25 ± 4	36 ± 3	38 ± 4	30 ± 5	28 ± 4	38 ± 6
C2, tip 3	37 ± 6	28 ± 5	22 ± 6	38 ± 3	38 ± 8	26 ± 6	22 ± 7	38 ± 5
C3, tip 1	30 ± 1	30 ± 1	30 ± 7	30 ± 1	31 ± 2	30 ± 2	28 ± 3	30 ± 1
C3, tip 2	31 ± 3	31 ± 2	24 ± 6	34 ± 4	34 ± 2	28 ± 6	23 ± 7	34 ± 5
C3, tip 3	34 ± 5	29 ± 5	21 ± 7	37 ± 5	33 ± 12	23 ± 8	17 ± 9	33 ± 7
C4, tip 1	31 ± 1	N. A.	25 ± 4	29 ± 1	35 ± 5	29 ± 9	22 ± 8	28 ± 2
C4, tip 2	30 ± 1	N. A.	19 ± 6	31 ± 3	31 ± 4	23 ± 12	18 ± 10	30 ± 8
C4, tip 3	29 ± 1	N. A.	16 ± 5	36 ± 4	31 ± 4	N. A.	N. A.	30 ± 9

(C1: 13%, random, C2: 50%, random; C3: 50%, self-organized; C4: 140% self-organized) and different AFM tips (tip1: ideal, tip2: sharpened, tip3: standard; see text for details).

Power spectrum (pow), minimum basis volume (min), Laplacian basis volume (lap) and *xy *projection (disk) methods were used. All values are in nanometers. Total number of deposited particles was approximately 50 (C1), 180 (C2, C3) and 400 (C4)

We can discuss each algorithm performance more in detail separately, sorted by the amount of user influence on measurement results (which itself can affect the method reliability significantly):

• Particle projection: particle projection evaluation method is in principle only as good as the segmentation used. Even if the watershed approach is very robust itself, in the presence of voids between particles, there is a need of fine tuning of the algorithm parameters in order to get an optimum segmentation. Moreover, this algorithm is the one most influenced by tip convolution effects as the particle projection changes significantly after convolution (namely for isolated particles).

• Particle volume--boundary minimum basis: here, the influence of the tip convolution is smaller as the volume of particle changes relatively less than its projection. However, the location of the proper minimum on the particle boundary is crucial here and for densely packed particles, this algorithm fails. Here, the tip is no more able to reach the substrate at the voids between particles and detected boundary minimum is wrong, which leads to a distortion, namely for larger probes. Moreover, it can be expected that this algorithm is significantly affected by the local substrate slope (even for isolated nanoparticles) as the substrate slope is not employed in the evaluation (see Figure 3c).

• Particle volume--Laplacian boundary interpolation basis: this approach treats the substrate geometry in an optimum way for isolated particles, and it can therefore provide slightly better results for high substrate roughnesses than the previous one. However, for densely packed particles or wide tips, it also fails, as the tip does not reach the substrate, similarly as in the previous case.

• Power spectrum analysis: as for area analysis methods, this approach fails for small coverages and non-ideal tips namely. As the tip convolution increases, the apparent width of the nanoparticles increases and there is no packing effect to block this. Resulting radii are much higher than would be expected. However, for dense packing this method seems to be quite robust, even for higher surface roughness. The main benefit of this method is that is does not use any segmentation; therefore, the amount of user influence is the smallest of all the discussed approaches.

As seen from the table, generally the particle volume -based methods are only suitable for small coverages, where they produce reasonable results even for rough substrates. With higher packing of particles or smaller AFM tip side slope, the tip cannot reach the void volume between particles, which leads to a loss of information. Volume or height analysis methods are most sensitive to this effect.

For power spectrum and particle projection methods, the densely packed particles are an ideal measurand. However, even for isolated nanoparticles, these methods can be very effective if the AFM tip is sharp enough.

For all the methods based on image segmentation, the effect of the roughness can be highly suppressed by using a proper segmentation technique. Thresholding can be effective only for an extremely small roughness or substrate curvature. With a robust implementation of any more complex technique, e.g., the watershed algorithm, the effect of substrate irregularities can be highly suppressed.

As a real example, the self-organized nanoparticle film presented in Figure 1d was analyzed using all the mentioned algorithms. The nominal particle diameter of NIST traceable polymer nanoparticles was (46 ± 2) nm, i.e., the particle radius was 23 nm. They were deposited on a flat silicon substrate forming a film of unknown thickness and measured using a standard AFM tip. The resulting radii were (23 ± 3) nm for the equivalent disk radius method (particle projection), (11 ± 8) nm for the minimum basis grain volume method, (8 ± 8) nm for the Laplacean basis grain volume method and (24 ± 2) nm for the power spectrum analysis method. We can see that similarly to the modeling results, the particle projection and power spectrum method provide significantly better results for this type of sample.

## Conclusion

In this article, the results of simulated nanoparticle measurements are presented. Nanoparticles are located on rough substrates, in some cases forming self-organized structures or even several layers. In this way, we simulate typical non-ideal conditions observed at nanoparticle measurement using atomic force microscopy. To treat different tip convolution effects, nanoparticles are convolved with several typical AFM tip geometries. Results of different nanoparticle analysis algorithms are compared and discussed.

It is shown that for isolated nanoparticles, height-based algorithms can be successful if the area below the nanoparticle is properly treated, both for flat and rough substrates, providing no systematic errors and uncertainties in the range of a few percents. However, for agglomerated nanoparticles or blunt AFM tips, these algorithms provide poor results and this effect is even worse for rough surfaces; in this case, the errors are comparable to estimated values.

For agglomerated particles, methods using lateral dimensions, both power spectrum -based and particle projection -based methods are very effective, even for rough substrates. These methods can provide results with uncertainty of a few percents and no systematic errors.

The worst case was observed for non-agglomerated nanoparticles with surface coverages between 30 ÷ 80%, where all classes of algorithms provide systematic errors and uncertainties larger than 10%. Here, a combination of all the approaches must be used and results must be interpreted very carefully.

It is shown that using a simple particle deposition modeling technique, together with a tip-sample convolution algorithm, one can get relatively easily estimates of uncertainty components related to data processing methods in nanoparticle analysis. This can be also understood as a fast approach for uncertainty estimation in any particular case in practice. The method described is implemented in the open source software package for SPM data analysis Gwyddion http://gwyddion.net.

## Competing interests

The authors declare that they have no competing interests.

## Authors' contributions

PK carried out nanoparticle simulations and statistical quantities evaluation and interpretation, MV performed atomic force microscopy measurements, DN wrote software routines for nanoparticle statistical analysis, OS and PD prepared nanoparticle samples. All authors read and approved the final manuscript.
